# Clinical Efficacy of Different Thoracoscopic Surgeries for Patients With Non-small Cell Lung Cancer

**DOI:** 10.3389/fsurg.2022.842047

**Published:** 2022-02-15

**Authors:** Tao Wang, Xi Liu, Lei Chen, Tao Liang, Xiaokuang Ning

**Affiliations:** Department of Thoracic Surgery, The First Medical Center of PLA General Hospital, Beijing, China

**Keywords:** non-small cell lung cancer, single-port thoracoscopic surgery, three-port thoracoscopic surgery, MMPs-7 mRNA, sMICA, T cell subsets

## Abstract

**Background:**

The aim of this study was to analyze the clinical efficacy of different thoracoscopic procedures in patients with non-small cell lung cancer and their correlation with matrix metalloproteinase-7 mRNA (MMPs-7 mRNA) and soluble major histocompatibility complex class I molecule A (sMICA), as well as their effect on T-cell subsets.

**Methods:**

A total of 100 patients with non-small cell lung cancer who received different thoracoscopic surgeries were divided into the Control group (three-port thoracoscopic surgery) and the study group (single-port thoracoscopic surgery). The two groups were evaluated to compare the perioperative indicators, MMPs-7 mRNA, sMICA expression levels, T-cell subsets, postoperative pain, complication rates, and prognostic outcomes at 1-year follow-up.

**Results:**

The operation time, blood loss, drainage tube placement time, incision length, and hospital stay in the study group were less than those in the control group (*P* < 0.05). There was no significant difference in the number of lymph node dissections between the two groups (*P* > 0.05). After 3 days, the expression levels of MMPs-7 mRNA and sMICA in the study group were lower than those in the control group (*P* < 0.05); CD4 +, CD8 +, and CD4 +/CD8 + in the study group were higher than those in the control group (*P* < 0.05). On days 1, 3, and 5, the visual analog score (VAS) of the study group was lower than that of the control group (*P* < 0.05); there was no significant difference in the complication rate between the two follow-up groups (*P* > 0.05), in which all patients completed the follow-up. After 1 year of follow-up, there was no significant difference in the tumor-free survival rate and overall survival rate between the two groups (*P* > 0.05).

**Conclusion:**

Compared with three-port thoracoscopic surgery, single-port thoracoscopic surgery can improve perioperative expression, shorten hospital stay, reduce serum tumor micrometastasis levels, improve immune metastasis mechanisms and reduce pain, which is of great significance to patients with non-small cell lung cancer. It is an effective, convenient, and safe surgical option that deserves wide clinical reference.

## Introduction

Lung cancer has become one of the malignant tumor diseases that seriously endanger human health and safety in China, and the incidence rate is increasing year by year. According to incomplete statistics, the incidence of lung cancer accounts for about 82% of the total incidence of lung cancer, with a high risk of death (1). At present, surgery, chemoradiotherapy, and other treatments are mostly advocated in clinical practice, of which surgery can play an ideal role in early cell lung cancer. With the development of medical technology in recent years, thoracoscopic surgery has been introduced, which has the advantages of less trauma, high safety, and rapid recovery, and has become the standard for cell lung cancer (2). In the past, three-hole thoracoscopic surgery was commonly used. Although it could achieve a certain effect, the incisions are large and significantly traumatic, with significant post-operative pain. With the continuous penetration of the minimally invasive concept, single-hole thoracoscopic surgery has emerged, fully reflecting the advantages of minimally invasive techniques (3). Metalloproteinase-7 mRNA (MMPs-7 mRNA), soluble major histocompatibility complex class I molecule A (sMICA), and T-cell subsets are correlated with lung cancer staging and prognosis. In view of this, this study analyzed the clinical efficacy of different thoracoscopic procedures for non-small cell lung cancer patients and their effects on MMPs-7mRNA, sMICA, and T-cell subsets to provide a theoretical basis for the subsequent clinical selection of the appropriate surgical approach.

## Materials and Methods

### General Information

This study was approved by the ethics committee of the Chinese People's Liberation Army (PLA) General Hospital. A total of 100 patients with non-small cell lung cancer admitted to our hospital from December 2018 to December 2019 were selected and treated with different thoracoscopic surgery for non-small cell lung cancer. They were divided into control group and study group according to different perforations, 50 cases for each group. By calculating the baseline data of the two groups, statistical analysis showed *p* > 0.05, which has a comparable value. Refer to Table 1 for details.

**Table 1:** The baseline data of the two groups.

	**Control group** **(***n*** = 50)**	**Study group** **(***n*** = 50)**	***X^2^/t-*** **value**	***P*** **-value**
Age (years)	63.15 ± 10.90	64.03 ± 11.18	0.399	0.691
Gender (male/female)	31/19	30/20	0.042	0.838
**TNM stage**				
Stage I	31	29	0.181	0.914
Stage II	13	14		
Stage III	6	7		
**Differentiation**				
Poorly differentiated	5	4	0.204	0.903
Moderately differentiated	36	35		
Well differentiated	9	11		

#### Inclusion Criteria

(1) All patients have been diagnosed with non-small cell lung cancer and meet the indications of video-assisted thoracoscopic surgery; (2) No metastasis was found by imaging examination before operation; (3) All patients have not received radiotherapy or chemotherapy before operation; (4) The postoperative tumor-node-metastasis (TNM) stage was I-III; (5) All patients signed informed consent and participated voluntarily.

#### Exclusion Criteria

(1) Those whose expected survival time was <6 months; (2) Those had severe mental illness history, Alzheimer's disease, or poor sense of self-cooperation; (3) Patients had severe heart, liver, and kidney insufficiency; (4) Those had coagulation dysfunction; (5) there is a history of other thoracic operations; (6) Patients were encountered other malignant tumors.

### Surgical Methods

The study group underwent single-hole thoracoscopic surgery for non-small cell lung cancer, guided patients to take healthy lateral positions, performed tracheal intubation general anesthesia, and confirmed one-lung ventilation after satisfactory anesthesia. An incision with a length of about 3 cm was made between the L4 or L5 ribs at the front axillary line, and the elastic rubber protective sleeve was placed in the hole as the operation hole. Then an incision with a length of about 1.5 cm was made between the L7 ribs at the back axillary line, and Trocar trocar was placed as the observation hole. Under the direct vision of the thoracoscope, the lung lobe (segment) was removed, and the hilar and mediastinal lymph nodes were dissected. The pathological specimens were sent for inspection, and the drainage tube was indwelling.

The control group received three-hole thoracoscopic non-small cell lung cancer surgery. Under the operation of the study group, an auxiliary operation hole was added, that is, an incision of about 2 cm in length was made in the L7 or L8 intercostal space of the subscapular corner line as the auxiliary operation hole, and exposure and traction were performed through the auxiliary operation hole. Under the direct thoracoscopic vision, the lobe (segment) was removed, and hilar and mediastinal lymph node dissection procedures were completed, the pathological specimen was submitted for examination, a drainage tube was indwelled, and the operation was completed.

### Observation Indicators

(1) We evaluated and compared the perioperative indicators of the two groups, and recorded the operation time, blood loss, drainage tube placement time, incision length, number of lymph node dissections, and hospital stay. (2) The expression of MMPs-7mRNA and sMICA were measured by RT-PCR and ELISA, respectively, before and 3 days after the operation. (3) T-cell subsets, CD4 +, CD8 +, and CD4+/CD8 + were measured and recorded by flow cytometry before and 3 days after the operation. (4) Visual analog scale (VAS) was used for postoperative pain degree, and the highest score was 10 points. The higher the score, the stronger the pain. (5) We record the postoperative complication rate of patients. (6) Prognostic results were followed up for 1 year. All patients were followed up throughout the whole process. The tumor-free survival rate and overall survival rate were counted after 1-year follow-up.

### Statistical Analysis

The data were analyzed by SPSS 22 software (IBM, Armonk, NY, USA), and the measurement data were tested by *t*-tests. The Chi-square test was used for counting data, and *P* < 0.05 was statistically significant.

## Results

### Comparison of Indicators Between the Two Groups

The operation time, blood loss, drainage tube placement time, incision length, and hospitalization time in the study group were less than those in the control group (*P* < 0.05). There was no significant difference in the number of lymph node dissections between the two groups (*P* > 0.05). Refer to Table 2 for details.

**Table 2:** Comparison of perioperative indexes between the two groups (X ± s, *n* = 50).

**Group**	**Operative Time (min)**	**Intraoperative blood loss (ml)**	**Drain Placement Time (d)**	**Incision length (cm)**	**Hospital stay (d)**	**Number of dissected lymph nodes (pieces)**
Study group	119.86 ± 9.12	124.65 ± 8.04	4.76 ± 1.02	4.19 ± 0.51	7.86 ± 1.37	13.45 ± 3.99
Control group	150.01 ± 11.36	158.11 ± 11.92	6.99 ± 1.13	10.08 ± 1.22	9.70 ± 1.25	14.70 ± 4.10
t	14.634	16.456	10.359	31.497	7.016	1.545
*P*	0.000	0.000	0.000	0.000	0.000	0.126

### Comparison of MMPs-7 MRNA and SMICA Expression Levels Between the Two Groups

On the 3rd day after the operation, the values of MMPs-7mRNA and sMICA in the study group were lower than those in the control group (*P* <0.05). Refer to Table 3 for details.

**Table 3:** Comparison of MMPs-7 mRNA and sMICA expression levels between the two groups ($\bar{\text{X}}$ ± s).

**Group**	***N***	**MMPs-7 mRNA**		**SMICA (pg/mL)**	
		**Pre-op**	**3 d after operation**	**Pre-op**	**3 d after operation**
Study group	50	29.30 ± 7.16	20.38 ± 4.49	382.61 ± 81.45	291.49 ± 65.28
Control group	50	30.15 ± 6.94	24.51 ± 6.40	379.22 ± 82.06	322.13 ± 74.50
t	-	0.603	3.736	0.207	2.187
*P*	-	0.548	0.000	0.836	0.031

### Comparison of T Cells Between the Two Groups

**Figure 1:**
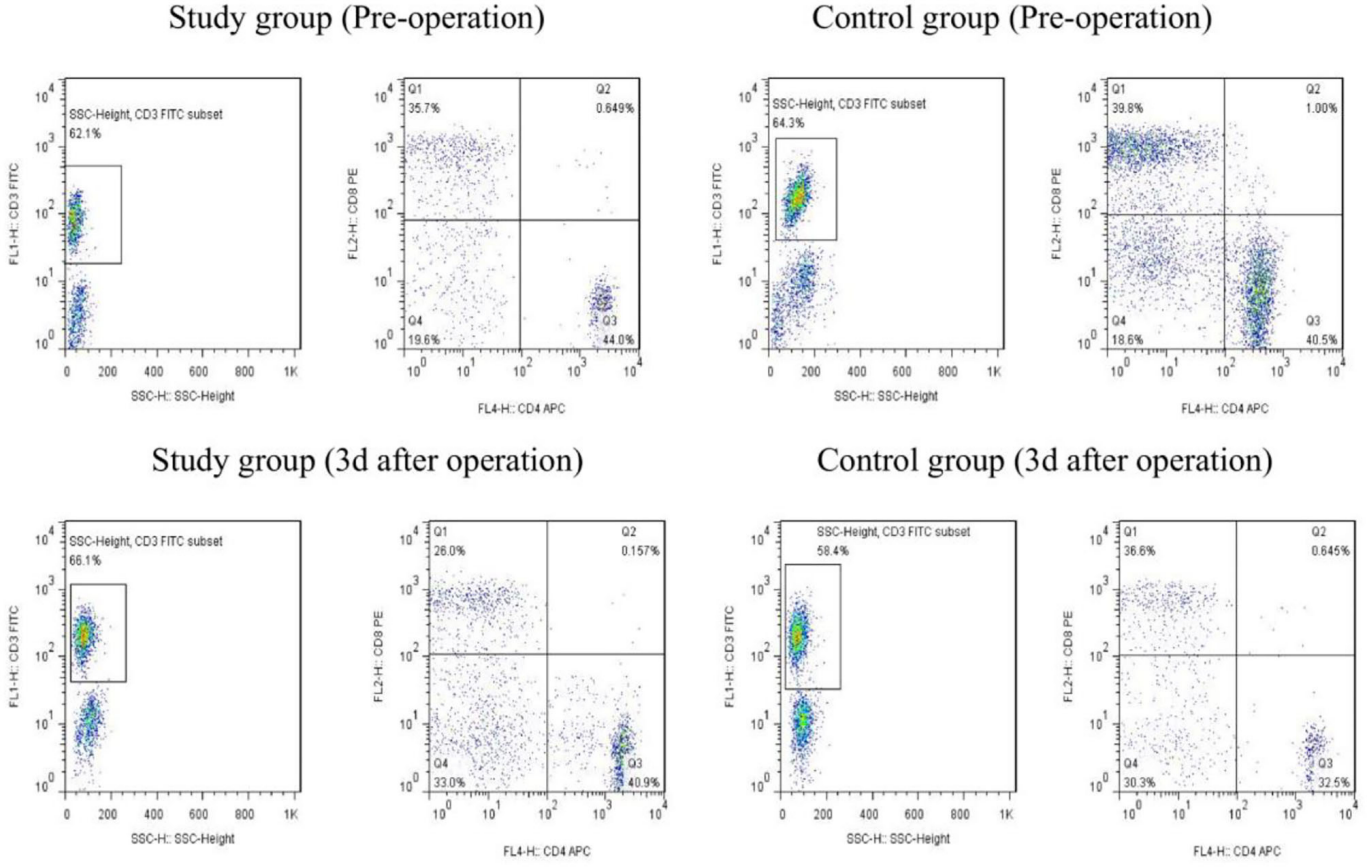
The changes of T-cell subsets (CD4 + and CD8 +) in the observation group and the control group were detected by flow cytometry before and 3 days after the operation.

The levels of CD4 +, CD8 +, and CD4+/CD8 + in the study group were higher than those in the control group on the 3rd day after the operation (*P* < 0.05). Refer to Table 4, Figure 1 for details.

**Table 4:** Comparison of T cell in the two groups ($\bar{\text{X}}$ ± s).

**Group**	***N***	**Operation time**	**CD4 + (%)**	**CD8 + (%)**	**CD4 +/CD8 +**
Study group	50	Pre-op	42.30 ± 2.89	38.65 ± 4.27	1.21 ± 0.23
		3d after operation	40.18 ± 4.16	27.70 ± 3.51	1.46 ± 0.19
Control group	50	Pre-op	41.65 ± 3.11	39.27 ± 3.30	1.22 ± 0.35
		3d after operation	32.23 ± 2.83	38.30 ± 2.40	0.96 ± 0.24
Intra-group comparison (*t/p*)	-	1.564/0.121	1.215/0.227	1.185/0.239
Comparison in control group (*t/p*)	-	10.964/0.000	12.078/0.000	4.332/0.000
Three days after operation, intra-group comparison (*t/p*)	-	7.097/0.000	8.980/0.000	4.620/0.000

### Comparison of VAS Score Between the Two Groups

On the 1st, 3rd, and 5th day after surgery, the VAS of the study group was lower than that of the control group (*P* < 0.05). Refer to Table 5 for details.

**Table 5:** Comparison of VAS scores between the two groups ($\bar{\text{X}}$ ± s, min).

**Group**	***N***	**1d after operation**	**3rd day after operation**	**5th day after operation**
Study group	50	3.09 ± 1.05	1.98 ± 0.70	1.02 ± 0.44
Control group	50	4.87 ± 1.42	3.26 ± 1.31	2.69 ± 1.05
t	-	7.127	3.094	10.373
*P*	-	0.000	0.000	0.000

### Comparison of Postoperative Complication Rate Between the Two Groups

There was no significant difference in the complication rate between the two groups (*P* > 0.05). Refer to Table 6 for details.

**Table 6:** Comparison of postoperative complication rate between the two groups [*n* (%)].

**Group**	***N***	**Wound infection**	**Atelectasis**	**Pneumothorax**	**Complication Rate**
Study group	50	2 (4.00)	1 (2.00)	1 (2.00)	4 (8.00)
Control group	50	3 (6.00)	2 (4.00)	0 (0.00)	5 (10.00)
X^2^	-	0.421	0.687	2.020	0.244
*P*	-	0.516	0.407	0.155	0.621

### Comparison of Prognostic Results Between the Two Groups After 1-Year Follow-Up

All patients were followed up for 1 year. There was no significant difference in tumor-free survival rate and overall survival rate between the two groups (*P* > 0.05). Refer to Table 7 for details.

**Table 7:** Comparison of 1-year follow-up outcomes between the two groups [n (%)].

**Group**	***N***	**Tumor free survival**	**Overall survival**
Study group	50	49 (98.00)	50 (10.00)
Control group	50	48 (96.00)	50 (100.00)
X^2^	-	0.687	-
*P*	-	0.407	-

## Discussion

Non-small cell lung cancer is characterized by high morbidity and high mortality, and minimally invasive surgery has become the first choice for the treatment of non-small cell lung cancer (4). It has been pointed out that for early-stage lung cancer patients, the clinical treatment effect of video-assisted thoracoscopic surgery is similar to that of traditional open-heart surgery, but video-assisted thoracoscopic surgery has the advantages of the clear and wide surgical field, small incision, low complication rate, and short recovery time, which are widely used in the treatment of lung cancer (5). In recent years, with the improvement, optimization, and development of video-assisted thoracoscopic techniques, three-hole video-assisted thoracoscopic lobectomy has achieved good results in the treatment of early-stage lung cancer. This procedure has the advantages of ease of operation, high visibility, and good exposure. However, the accessory surgical hole was found to be in a special location between the L7 or L8 ribs in the subscapularis angle line. This area is distributed with many muscle groups and has a small intercostal space, making it prone to intraoperative bleeding and injury, as well as postoperative pain in the posterior chest wall (6–8). In addition, the length of the three-hole incision is large, which will undoubtedly form postoperative scars, thus affecting the aesthetics. With the development of minimally invasive technology and increasing the research on non-small cell lung cancer, single-hole thoracoscopic lobectomy was born. As early as 2005, some studies clearly pointed out that single-hole thoracoscopic lobectomy for early lung cancer has high feasibility and safety (9). After research and analysis, the main reason is that the operation is done at L4 or L5 intercostal space in the axillary front line, where the intercostal space is relatively wide and the muscle group is not abundant, so the risk of bleeding and injury is reduced to a certain extent (10). The operation time and the incision length of single-hole thoracoscopic surgery are short, and it is difficult to form obvious scars after the operation, which meets the aesthetic requirements of patients. In addition, the operation is a single-hole operation, which can effectively avoid muscle injury and nerve damage caused by the auxiliary operation hole, speed up the postoperative rehabilitation progress, and shorten the hospitalization time (11).

However, no matter what kind of operation, it will cause a stress reaction of the body, which will lead to the decline of immune function, thus prone to infection and tumor micrometastasis. Especially for tumor patients, immune function can determine the spread, recurrence, and recovery progress of tumors after the operation (12). Therefore, reducing the stress response and improving immunity are particularly critical to reducing the level of tumor micrometastasis factors.

This study shows that the operation time, blood loss, drainage tube placement time, incision length, and hospitalization time of the study group were less than those of the control group. It is suggested that compared with three-hole thoracoscopic surgery, single-hole thoracoscopic surgery for non-small cell lung cancer has a shorter operation time, can reduce intraoperative bleeding, ensure early extraction of the drainage tube, and has shorter incision length, which is beneficial to rapid healing of the incision and less hospital stay. There was no significant difference in the number of lymph node dissections between the two groups, suggesting that the number of lymph node dissections in three-hole thoracoscopic surgery was similar to that in single-hole thoracoscopic surgery. According to the data, postoperative tumor micrometastasis will be affected by extracellular matrix remodeling, immune mechanism, and other aspects, and has a close relationship with the prognosis of patients with non-small cell lung cancer (13, 14). Through tumor cytology examination, MMP-7 was highly expressed in patients with tumor (15). During the apoptosis of tumor cells, MICA protein on the cell surface falls off and finally forms sMICA, which can hinder the killing activity of T cell subsets (CD8 +) (16). Three days after the operation, the values of MMPs-7mRNA and sMICA in the study group were lower than those in the control group, suggesting that the expression level of serum tumor micrometastasis factor can be reduced by single-port thoracoscopic surgery for non-small cell lung cancer, thus ensuring the improvement of tumor metastasis microenvironment. It has been reported that T lymphocyte subsets can accurately reflect cellular immune function (17). CD4 +, CD8 +, and CD4+/CD8 + in the study group were higher than those in the control group, suggesting that single-port thoracoscopic surgery for non-small cell lung cancer can effectively protect cellular immune function. The main reason is that single-hole thoracoscopic surgery has a short operation time and less bleeding, which can obviously reduce the impact on the immune mechanism of the body (18). In addition, this operation has less trauma, and at the same time, it can effectively preserve the integrity of the thorax, avoid muscle and nerve damage, and has a low-stress response, which can promote the immunity to return to normal (19, 20). On the 1st, 3rd, and 5th day after the operation, the VAS of the study group was lower than that of the control group, suggesting that the application of single-hole thoracoscopic surgery for non-small cell lung cancer can reduce the postoperative pain, relieve its pain and improve the surgical tolerance and compliance. There is no significant difference in complication rate between the two groups, suggesting that three-hole/single-hole thoracoscopic surgery for non-small cell lung cancer is not easy to cause postoperative complications such as incision infection, atelectasis, and pneumothorax, and the operation is safe. All patients were followed up for 1 year. There was no significant difference in tumor-free survival rate and overall survival rate between the two groups, suggesting that there was no significant difference in the influence of different thoracoscopic surgery on the 1-year survival rate after surgery. However, in this study, the number of samples is small, the follow-up time is short, and there is no comparison of long-term recurrence and metastasis of single-hole/three-hole thoracoscopic surgery for non-small cell lung cancer. In the future, it is necessary to expand the sample size and extend the follow-up time to further confirm the practical application value of the above two operations.

## Conclusions

In conclusion, compared with three-port thoracoscopic surgery, single-port thoracoscopic surgery in patients with non-small cell lung cancer can optimize perioperative indicators, shorten hospital stay, reduce the expression level of serum tumor micrometastatic factors, improve immune mechanisms, and be less painful. It is of great significance for patients with non-small cell lung cancer and is an effective, convenient, and safe surgical option, which is worthy of extensive clinical reference.

## Data Availability Statement

The original contributions presented in the study are included in the article/supplementary material, further inquiries can be directed to the corresponding authors.

## Ethics Statement

The studies involving human participants were reviewed and approved by the Ethics Committee of PLA General Hospital. The patients/participants provided their written informed consent to participate in this study.

## Author Contributions

TW and XL designed the study and prepared the manuscript. LC and TL collected the data. TW, XL, and XN analyzed the data. All authors read and approved the final manuscript.

## Conflict of Interest

The authors declare that the research was conducted in the absence of any commercial or financial relationships that could be construed as a potential conflict of interest.

## Publisher's Note

All claims expressed in this article are solely those of the authors and do not necessarily represent those of their affiliated organizations, or those of the publisher, the editors and the reviewers. Any product that may be evaluated in this article, or claim that may be made by its manufacturer, is not guaranteed or endorsed by the publisher.
